# Appendectomy and Risk of Nonyphoidal Salmonella Infection in Children

**DOI:** 10.1001/jamanetworkopen.2025.55278

**Published:** 2026-01-23

**Authors:** Jyun-Yi Guo, Wei-Szu Lin, Ching-Heng Lin, Meng-Che Wu

**Affiliations:** 1 Division of Pediatric Gastroenterology, Children’s Medical Center, Taichung Veterans General Hospital, Taichung, Taiwan; 2 Department of Medical Research, Taichung Veterans General Hospital, Taichung, Taiwan; 3 Department of Post-Baccalaureate Medicine, College of Medicine, National Chung Hsing University, Taichung; 4 School of Medicine, Chung Shan Medical University, Taichung, Taiwan

## Abstract

**Importance:**

The appendix is thought to have immunological functions. However, its role in protecting against enteric infections such as nontyphoidal Salmonella (NTS) remains unclear in children.

**Objective:**

To investigate whether pediatric appendectomy is associated with an increased hazard of subsequent NTS infection.

**Design, Setting, and Participants:**

This was a nationwide population-based cohort study using data from Taiwan’s National Health Insurance Research Database between January 1, 2000, and December 31, 2019. Children who underwent appendectomy were matched to children without appendectomy in a 1:4 ratio based on age, sex and index date. Data were analyzed from January 2000 to December 2019.

**Exposure:**

Appendectomy during childhood.

**Main Outcomes and Measures:**

The primary outcome was the incidence of NSA infection, defined as 3 outpatient or 1 inpatient diagnosis after the index date. The hypothesis that appendectomy increases susceptibility to NTS infection was specified before data collection. Cox proportional hazards models were used to estimate adjusted hazard ratios (aHRs).

**Results:**

The cohort included 18 654 children who underwent appendectomy (mean [SD] age, 10.04 (4.17) years; 11 883 [63.7%] male) and 74 616 matched children in the control group (mean [SD] age, 9.73 [4.30] years; 47 532 [63.7%] male). Children who underwent appendectomy had a significantly higher risk of NTS infection compared with children in the control group (aHR, 1.58; 95% CI, 1.17-2.13). The risk was pronounced in children younger than 5 years (aHR, 2.00; 95% CI, 1.35-2.97). Regarding follow-up periods, the risk was elevated but not statistically significant for 1 to 4 years (aHR, 1.51; 95% CI, 0.98-2.32) and 5 or more years (aHR, 1.78; 95% CI, 0.89-3.54).

**Conclusions and Relevance:**

In this nationwide cohort study of children in Taiwan, pediatric appendectomy was associated with an increased hazard of NTS infection in the overall population and in particular among children younger than 5 years. These findings suggest a potential need for surveillance and preventive strategies in children following appendectomy, but further studies are warranted to develop and evaluate specific interventions.

## Key Points

**Question:**

Is undergoing appendectomy associated with increased risk of developing nontyphoidal Salmonella (NTS) infection in children?

**Findings:**

In this nationwide cohort study in Taiwan involving 18 654 children who had an appendectomy and 74 616 children in a matched control group, children who underwent appendectomy had a 1.58-fold higher risk of developing NTS infection compared with the control group.

**Meaning:**

These findings suggest that appendectomy is associated with a significantly increased risk of future NTS infection in children, suggesting the appendix may play a protective role in gut immunity.

## Teaser

This cohort study evaluates whether pediatric appendectomy is associated with an increased hazard of subsequent nontyphoidal Salmonella infection.

## Introduction

The human appendix is a small, tubelike structure that extends from the cecum into the beginning of the large intestine.^1^ Appendectomy continues to be one of the most frequently performed emergency surgical procedures. The incidence of appendectomy in Western countries is approximately 26 to 100 per 100 000 person-years. Epidemiological data indicate that it most frequently occurs during the second decade of life.^2,3,4,5^ Several studies have indicated that the appendix functions similarly to other gut-associated lymphoid tissues (GALT).^6,7,8,9^ The immune cell population identified in the mucosa of the appendix includes macrophages and a large number of immunoglobulin-producing plasma cells.^10^ The appendix may be associated with bacteria in the mammalian gut and is thought to provide a suitable environment for the growth of commensal bacteria.^1,11^ The microbiota of the human appendix exhibits significant individual variation.^12^ Although numerous studies have examined the composition of the intestinal microbiome, there are limited data available specifically for the appendix. Since the appendix contains lymphoid cells that contribute to immune responses, its removal can potentially impact immune function. Nontyphoidal Salmonella (NTS) is a significant cause of infectious diarrhea worldwide.^13^ NTS infections can indeed lead to severe invasive bacteremia and disseminated disease, particularly in vulnerable populations.^14,15^ Salmonella employs several mechanisms to interfere with B cell activity, including IgA-producing plasma cells.^16^ Studies have found correlations between a history of appendectomy and a higher risk of certain infections, including recurrent Clostridium difficile infection, liver abscess, and sepsis.^17,18,19,20^ The potential association between appendectomy and NTS infection remains unclear. We hypothesize that appendectomy may increase gut susceptibility to NTS infections. This study aims to explore the association between appendectomy and the risk of developing NTS infections in children.

## Methods

### Data Source

The Taiwan National Health Insurance Research Database (NHIRD) is a comprehensive, population-based claims database covering over 99% of Taiwan’s population since the inception of the National Health Insurance program in 1995. The NHIRD contains detailed sociodemographic, diagnostic, procedural, and prescription data, and is widely recognized for its utility in large-scale epidemiological and health services research.^21,22^ Claims data from January 1, 2000, to December 31, 2019, were included in the study. Numerous studies have leveraged the NHIRD for population-based investigations.^23,24,25^ Validation studies have confirmed the accuracy of major disease diagnoses and medication records within the NHIRD, supporting its reliability for research purposes. However, limitations such as potential misclassification, unmeasured confounding, and variable coding practices across institutions must be considered. Methodological advancements, including code validation and algorithm sharing, have improved data quality and interstudy comparability.^21^

This study was approved by the institutional review board of Taichung Veterans General Hospital. The requirement for informed consent was waived because all data from the NHIRD were anonymized and deidentified. This study was designed and reported in accordance with the Strengthening the Reporting of Observational Studies in Epidemiology (STROBE) reporting guidelines for cohort studies. Patient consent was waived because all data from the NHIRD were anonymized and delinked from personal information before being made available to the researchers.

### Participant Selection

The study initially screened participants from a large sample, with a total population of 3 880 193 newborns born between 2000 and 2019. After excluding children with malignant neoplasm, there were 3 860 175 children in this study. The participants were then divided into 2 groups: a control group and an appendectomy group. Appendectomy was identified using health insurance procedure codes 74004B, 97201K, 97202A, 97203B, and 74002B. To reduce potential reverse causality and perioperative bias, children who developed NTS infection within 3 months of the index appendectomy date were excluded. Children who underwent appendectomy were assigned to the appendectomy group, while those in the control group without appendectomy were identified from the same database. Each appendectomy case was matched to 4 children in the control group in a 1:4 ratio based on age, sex, and index date to ensure comparability between groups. The control group included participants under the age of 18 who did not undergo an appendectomy, while the appendectomy group consisted of those who had undergone the procedure. The index date was defined as the specific operation day on which each individual underwent an appendectomy. Family income and urbanization levels were obtained from data within the Taiwan National Health Insurance program. Given that all data were sourced from NHIRD records, the participation rate of study participants was effectively complete.

### Covariates and Matching

To ensure comparability between the control and appendectomy groups, covariate matching was applied. Analyses were performed using a complete-case approach. A total of 425 463 newborns were excluded at the initial stage due to incomplete baseline information (missing data on sex, place of residence, or family income). Participants were stratified into 4 age groups (<5, 5-9, 10-14, and ≥15 years). Age distributions were identical between the control and appendectomy groups. Sex distribution was also balanced between the 2 groups, with no significant difference. Family income in New Taiwan dollars was divided into 4 levels ($≤18 780, $18 781-27 600, $27 601-42 000, and $>42 000). Participants were classified based on urbanization levels of their residence (urban, suburban, and rural). Additionally, to control for potential health status differences, we analyzed the distribution of comorbidities recorded before the index date, including autism (*International Classification of Diseases, Ninth Revision, Clinical Modification (ICD-9-CM)* code 299 and *ICD-10-CM* code F84), asthma (*ICD-9-CM* code 493 and *ICD-10-CM* code J44.J45), atopic dermatitis (*ICD-9-CM* code 691 and *ICD-10-CM* code L20.L22), diabetes mellitus (*ICD-9-CM* code 250 and *ICD-10-CM* code E10.E11.E13), congenital heart anomaly (*ICD-9-CM* code 745-747 and *ICD-10-CM* code Q20-28), chronic kidney disease (*ICD-9-CM* code 580-589 and *ICD-10-CM* code N00-08,N17-19), congenital gastrointestinal anomaly disease (*ICD-9-CM* code 750-751 and *ICD-10-CM* code Q38-45), chronic liver diseases (*ICD-9-CM* code 070. 570-576 and *ICD-10-CM* codes B15-19, K70-77, K 80-83, K87, and K91.5), constipation (*ICD-9-CM* code 564 and *ICD-10-CM* code K59), and cerebral palsy (*ICD-9-CM* code 343 and *ICD-10-CM* code G80).^21,26,27^ These covariates matching and analysis results support the robustness of the statistical comparisons between the 2 groups.

### Statistical Analysis

Statistical analyses were conducted between January 1, 2000, to December 31, 2019. Baseline characteristics between the control and appendectomy groups were analyzed using descriptive statistics, with categorical variables expressed as counts and percentages. Differences between the groups were assessed using χ^2^ tests for categorical variables. To identify factors associated with NTS infection (*ICD-9-CM* code 003, *ICD-10-CM* code A02, and 3 outpatient diagnoses or 1 inpatient diagnosis), Cox proportional hazards regression analyses were conducted. We present both crude and adjusted hazard ratios (aHR) with 95% CIs and *P* values for each variable. Initial crude HRs were calculated without adjusting for other variables, providing a baseline association for each characteristic with the risk of NTS infection. aHRs were estimated by including all variables in a multivariable model to control for potential confounders. Significant factors were identified with a 2-sided *P* value less than .05. We additionally performed stratified Cox proportional hazards analyses by follow-up period, age, sex, family income, and urbanization. We also conducted a supplementary conditional Cox proportional hazards regression stratified by matched sets to assess whether incorporating the matching structure was associated with variance estimates or altered the magnitude of the association between appendectomy and NTS infection (eTables 1 and 2 in Supplement 1). Kaplan-Meier survival curves were constructed to compare the cumulative incidence of NTS infection between children with and without appendectomy, and the log-rank test was used to assess differences between groups. Statistical analyses were performed using SAS version 9.4 (SAS Institute). Data were analyzed from January 2000 to December 2019.

## Results

**Figure 1.**
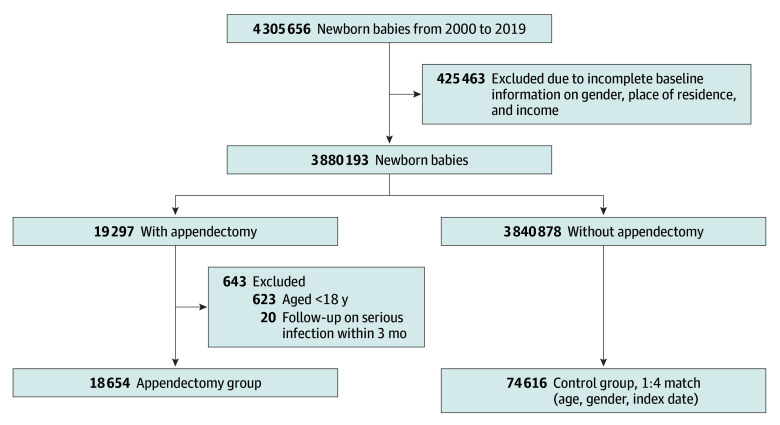
Flowchart of Participant Recruitment

The study flowchart is shown in Figure 1. After excluding participants with malignant neoplasms, 3 860 175 children remained from the initial 3 880 193 newborns identified. Of these, 18 654 were assigned to the appendectomy group (mean [SD] age, 10.04 (4.17) years; 11 883 [63.7%] male) and 74 616 to the matched control group (mean [SD] age, 9.73 [4.30] years; 47 532 [63.7%] male) (Table 1). The appendectomy group had slightly lower mean (SD) family income ($23 041 [$15 556] vs $24 122 [$15 952]) and a higher proportion residing in rural areas (5779 participants [31.0%] vs. 21 857 participants [29.3%]). Comorbidities such as asthma, atopic dermatitis, congenital gastrointestinal anomalies, chronic liver disease, and constipation were more common in the appendectomy group, while autism and cerebral palsy showed no differences. Antibiotic exposure within 1 year before the index date was also higher in the appendectomy group (11 111 participants [59.6%] vs. 34 689 [46.5%]).

**Table 1.** Baseline Characteristics of Study Participants

Characteristic	Participants, No. (%)			*P* value
	Total No.	Control group (n = 74 616)	Appendectomy group (n = 18 654)	
Age, mean (SD), y	NA	9.73 (4.30)	10.04 (4.17)	
<5	10 880	8704 (11.7)	2176 (11.7)	>.99
5-9	35 825	28 660 (38.4)	7165 (38.4)	
10-14	33 260	26 608 (35.7)	6652 (35.7)	
≥15	13 305	10 644 (14.3)	2661 (14.3)	
Sex				
Female	33 855	27 084 (36.3)	6771 (36.3)	>.99
Male	59 415	47 532 (63.7)	11 883 (63.7)	
Family income, NT$				
Mean (SD)	NA	24 122.40 (15 951.81)	23 040.83 (15 555.62)	NA
≤18 780	28 452	22 512 (30.2)	5940 (31.8)	<.001
18 781-27 600	36 173	28 724 (38.5)	7449 (39.9)	
27 601-42 000	18 808	15 297 (20.5)	3511 (18.8)	
>42 000	9837	8083 (10.8)	1754 (9.4)	
Urbanization				
Urban	52 295	42 165 (56.5)	10 130 (54.3)	<.001
Suburban	13 339	10 594 (14.2)	2745 (14.7)	
Rural	27 636	21 857 (29.3)	5779 (31.0)	
Comorbidity				
Autism	815	663 (0.9)	152 (0.8)	.333
Asthma	25 493	20 166 (27.0)	5327 (28.6)	<.001
Atopic dermatitis	21 123	16 795 (22.5)	4328 (23.2)	.04
Congenital heart anomaly	2585	2022 (2.7)	563 (3.0)	.02
Congenital gastrointestinal anomaly disease	923	695 (0.9)	228 (1.2)	<.001
Chronic liver diseases	978	739 (1.0)	239 (1.3)	.001
Constipation	17 786	13 286 (17.8)	4500 (24.1)	<.001
Cerebral palsy	305	242 (0.3)	63 (0.3)	.77
Antibiotic (before 1 y)				
No	47 470	39 927 (53.5)	7543 (40.4)	<.001
Yes	45 800	34 689 (46.5)	11 111 (59.6)	

Abbreviations: NA, not applicable; NT, New Taiwan.

**Figure 2.**
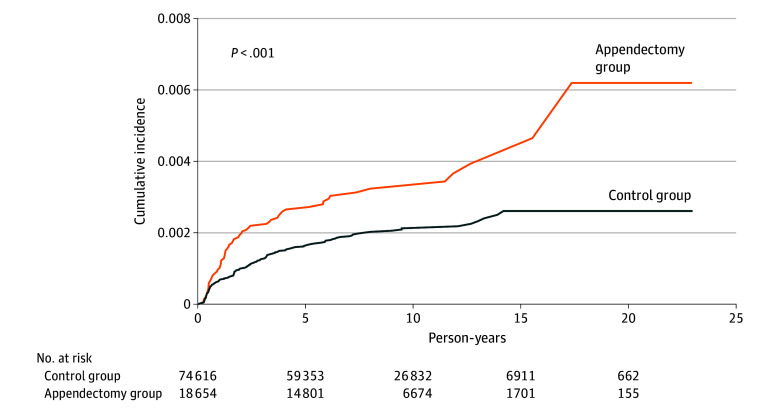
Kaplan-Meier Analysis of Cumulative Incidence of Nontyphoidal Salmonella Between Appendectomy and Control Groups

The Kaplan-Meier curves are shown in Figure 2. The cumulative incidence of NTS infection was significantly higher in the appendectomy group compared with the control group. The log-rank test for comparing the cumulative incidence curves yielded a *P* value of 001.

In the univariable and multivariable analysis (Table 2), we reported both crude and aHRs for appendectomy and all covariates, enabling readers to evaluate univariable associations and the outcomes of multivariable adjustment. Children in the appendectomy group had a significantly increased risk of NTS infection, with an aHR of 1.58 (95% CI, 1.17-2.13; *P* = .003). Effect sizes were greatest among children under 5 years old relative to those aged 15 years or older (aHR, 14.88; 95% CI, 6.88-32.18). The risk for children aged 5 to 9 years was also elevated (aHR, 2.56; 95% CI, 1.17-5.62), though to a lesser extent. This suggests that younger children are at a notably greater risk for infection as reflected in the higher HR observed in this age group. Male children had a higher risk than female children (aHR, 1.40; 95% CI, 1.04-1.89; *P* = .03). No significant gradient was observed with increasing family income. There was little difference in risk based on urbanization. For comorbidities, atopic dermatitis was significantly associated with an increased risk of NTS infection, with an aHR of 1.46 (95% CI, 1.08-1.98; *P* = .02). However, other comorbidities were not significantly associated with the risk of infection after adjustment. These results suggest that appendectomy and specific demographic and health characteristics may increase susceptibility to NTS infection. Prior antibiotic use within 1 year before the index date was also associated with increased risk (aHR, 1.47; 95% CI, 1.09-1.97; *P* = .01).

**Table 2.** Univariable and Multivariable Analysis of Factors Associated Nontyphoidal Salmonella Infection

Characteristic	Crude HR (95% CI)	*P* value	Adjusted HR (95% CI)	*P* value
Appendectomy operation group				
No	1 [Reference]	NA	1 [Reference]	NA
Yes	1.65 (1.23-2.23)	.001	1.58 (1.17-2.13)	.003
Age, y				
<5	15.63 (7.26-33.64)	<.001	14.88 (6.88-32.18)	<.001
5-9	2.65 (1.21-5.80)	.02	2.56 (1.17-5.62)	.02
10-14	1.40 (0.61-3.21)	.43	1.41 (0.61-3.23)	.42
≥15	1 [Reference]	NA	1 [Reference]	NA
Sex				
Female	1 [Reference]	NA	1 [Reference]	NA
Male	1.35 (1.00-1.82)	.047	1.40 (1.04-1.89)	.03
Family income, NT$				
≤18 780	1 [Reference]	NA	1 [Reference]	NA
18 781-27 600	1.06 (0.77-1.46)	.73	0.99 (0.72-1.37)	.96
27 601-42 000	0.88 (0.59-1.31)	.52	0.83 (0.55-1.25)	.37
>42 000	0.93 (0.56-1.53)	.77	0.84 (0.51-1.40)	.50
Urbanization				
Urban	1 [Reference]	NA	1 [Reference]	NA
Suburban	1.13 (0.76-1.67)	.56	1.07 (0.72-1.60)	.73
Rural	1.12 (0.83-1.52)	.46	1.10 (0.81-1.50)	.54
Comorbidity				
Asthma				
No	1 [Reference]	NA	1 [Reference]	NA
Yes	0.73 (0.53-1.02)	.07	0.95 (0.67-1.35)	.77
Atopic dermatitis				
No	1 [Reference]	NA	1 [Reference]	NA
Yes	1.42 (1.05-1.91)	.02	1.46 (1.08-1.98)	.02
Congenital heart anomaly				
No	1 [Reference]	NA	1 [Reference]	NA
Yes	0.69 (0.26-1.85)	.46	0.67 (0.25-1.81)	.43
Congenital gastrointestinal anomaly disease				
No	1 [Reference]	NA	1 [Reference]	NA
Yes	0.97 (0.24-3.89)	.96	0.79 (0.19-3.19)	.73
Chronic liver diseases				
No	1 [Reference]	NA	1 [Reference]	NA
Yes	0.96 (0.24-3.85)	.95	1.20 (0.30-4.87)	.80
Constipation				
No	1 [Reference]	NA	1 [Reference]	NA
Yes	0.90 (0.63-1.29)	.56	1.01 (0.70-1.45)	.98
Antibiotic (before 1 y)				
No	1 [Reference]	NA	1 [Reference]	NA
Yes	2.02 (1.52-2.70)	<.001	1.47 (1.09-1.97)	.01

Abbreviations: HR, hazard ratio; NA, not applicable; NT, New Taiwan.

Table 3 shows a multivariable Cox proportional hazards regression analysis stratified by follow-up periods, age, sex, family income, and urbanization. Children under 5 years of age had a higher risk of NTS infection in the appendectomy group (aHR, 2.00; 95% CI, 1.35-2.97; *P* < .001), and those aged 15 years or older had an elevated but not statistically significant risk (aHR, 1.92; 95% CI, 0.36-10.10). Regarding follow-up periods, the risk was elevated but not statistically significant for 5 or more years (aHR, 1.78; 95% CI, 0.89-3.54; *P* = .10) and for 1 to 4 years (aHR, 1.51; 95% CI, 0.98-2.32; *P* = .06), while during the first year postappendectomy, the HR was lower and not statistically significant (aHR, 0.56; 95% CI, 0.27-1.14; *P* = .11). Male children in the appendectomy group were significantly more likely to develop NTS infection compared with female children (aHR, 1.60; 95% CI, 1.12-2.30; *P* = .01). Lower family income levels were associated with a higher risk of NTS infection or death in both groups. Individuals with an income of $18 781 to $27 600 had a significantly higher risk (aHR, 2.18; 95% CI, 1.41-3.39; *P* = .001) compared with those with higher incomes. Rural participants in the appendectomy group also had a significantly higher risk (aHR, 1.83; 95% CI, 1.09-3.06; *P* = .02).

**Table 3.** Multivariable Analysis of Factors Associated Nontyphoidal Salmonella Infection, Stratified by Follow-Up Periods, Age, Sex, Family Income, and Urbanization

Characteristic	Adjusted HR (95% CI)	*P* value	***P *value for interaction**
Age, y			
<5	2.00 (1.35-2.97)	.001	.15
5-9	1.23 (0.68-2.21)	.49	
10-14	0.97 (0.39-2.43)	.95	
≥15	1.92 (0.36-10.10)	.44	
Follow-up periods, y			
<1	0.56 (0.27-1.14)	.11	.09
1-4	1.51 (0.98-2.32)	.06	
≥5	1.78 (0.89-3.54)	.10	
Sex			
Female	1.50 (0.86-2.62)	.16	.93
Male	1.60 (1.12-2.30)	.01	
Family income, NT$			
≤18 780	1.34 (0.77-2.32)	.30	.67
18 781-27 600	2.18 (1.41-3.39)	.001	
27 601-42 000	0.97 (0.42-2.25)	.94	
>42 000	1.22 (0.41-3.68)	.72	
Urbanization			
Urban	1.46 (0.95-2.23)	.09	.52
Suburban	1.43 (0.67-3.06)	.35	
Rural	1.83 (1.09-3.06)	.02	

Abbreviations: HR, hazard ratio; NT, New Taiwan.

## Discussion

This longitudinal population cohort study found a significant association between appendectomy and an increased risk of NTS infection. In this study, appendectomy was associated with a 1.58-fold increased risk of future NTS infection in children. In the multivariable analyses, individuals in the appendectomy group generally showed an increased hazard of NTS infection compared with the control group, reaching statistical significance in the younger age cohorts. This finding supports the hypothesis that the appendix may play a protective role in immune function, with its removal potentially increasing susceptibility to certain infections. These findings underscore the importance of implementing targeted preventive strategies and monitoring to reduce infection risk in susceptible populations.

The appendix is abundant in lymphatic tissue and plays a crucial role in regulating immune responses. A high proportion of B cells enzymatically dissociated from the human appendix are primarily committed to the IgA isotype.^28^ The appendix may function as a microbial reservoir aiding in the repopulation of the gastrointestinal tract when necessary.^5,10,12,28^ The human appendix is increasingly recognized as a reservoir for beneficial gut microbiota, hosting diverse microorganisms such as Eubacterium rectale, members of the Firmicutes and Bacteroidetes phyla, Actinobacteria, Faecalibacterium prausnitzii, Proteobacteria species, and Akkermansia muciniphila.^29^ There is considerable diversity and interindividual variability in the microbial composition of the appendix.^12^ IgA neutralizes pathogens at mucosal surfaces by binding antigens and preventing epithelial adhesion. Specifically, secretory IgA limits Salmonella pathogenicity island 1 type III secretion system-mediated epithelial invasion and effector protein translocation.^30^ Most reports on appendectomy and the subsequent increased risk of disease primarily focus on adults.^31,32,33^ Our study, for the first time we know of, examined the association of appendectomy on the risk of NTS infection in a pediatric population. Recent studies have reported that appendectomy is associated with an increased risk of developing certain clinical diseases. These conditions include the subsequent development of colorectal cancer,^33,34^ an elevated risk of ischemic heart disease,^31^ and an increased likelihood of rheumatoid arthritis,^35^ all of which have been significantly linked to gut dysbiosis. There is also evidence suggesting that an appendectomy performed for appendicitis may be significantly associated with an increased risk of developing Crohn’s disease.^36^ This association raises questions about the role of the appendix and its removal in gut health and immune regulation. Appendectomy has been shown to disrupt this balance, reducing the abundance of health-promoting bacterial populations. Alterations in microbiome composition following appendectomy may result in a reduction of butyrate-producing bacteria, potentially contributing to these health risks.^37^ Butyrate’s role in strengthening tight junctions and upregulating antimicrobial peptides is well documented, and its loss may facilitate NTS translocation across the intestinal barrier.^38,39,40^ In our study, additionally, age emerged as a factor associated with risk of infection, with children under 5 years showing an especially elevated risk. This elevated risk may reflect age-related vulnerabilities in immune function and exposure patterns. Murine studies show appendiceal lymphoid tissue is essential for priming naive B cells to undergo IgA class-switch recombination via TGF-β and retinoic acid signaling. Early appendectomy may disrupt this process, delaying mucosal immunity maturation.^41^ Furthermore, atopic dermatitis was significantly associated with a higher risk, potentially indicating a link between immune dysregulation in atopic conditions and susceptibility to infection.

Many years ago, pediatric surgeons became more cautious about performing incidental appendectomy during unrelated abdominal procedures, particularly in children with congenital urologic or bowel conditions. The appendix is often used as a conduit in reconstructive procedures such as the Malone antegrade continence enema and catheterizable urinary or fecal diversion channels, making preservation critical in selected patients.^42,43^ This change reflects a careful risk benefit analysis, weighing potential future utility against the risk of appendicitis. Accumulating evidence has also underscored the immunologic role of the appendix, including contributions to mucosal immunity and serving as a reservoir for commensal gut microbiota. Such insights are now routinely incorporated into preoperative counseling, highlighting possible long-term consequences of removal. In parallel, management of uncomplicated pediatric appendicitis has shifted. Large multi-institutional studies and meta-analyses demonstrate that nonoperative management with antibiotics is a safe and effective alternative to surgery in carefully selected children.^44,45^ Compared with appendectomy, antibiotic therapy is associated with fewer disability days, similar complication rates, and high parental satisfaction.^46^ Taken together, these developments highlight the importance of individualized, evidence-based decision-making. Current considerations extend beyond the immediate risks and benefits of appendectomy vs antibiotics to include the potential future surgical utility of the appendix and its immunologic functions. This comprehensive approach is now reflected in contemporary counseling and research efforts aimed at optimizing outcomes for children with appendicitis.

We also accounted for socioeconomic status and residential area as potential confounding factors. The baseline differences suggest that socioeconomic factors and specific health conditions may influence the likelihood of undergoing an appendectomy. The aHR was highest during the 5-year follow-up period (aHR, 1.78), possibly indicating that the increased susceptibility to infection may lessen over time as the body adapts to the loss of the appendix or compensatory immune mechanisms develop. Interventions tailored to these high-risk groups could help mitigate infection risks, potentially involving enhanced hygiene education, timely vaccinations, or targeted follow-up care.

### Strengths and Limitations

This study had several strengths. We used data from a nationwide database, which offered a larger, population-based sample compared with previous studies. Additionally, the longitudinal design allowed for stronger evidence regarding the association between appendectomy and the risk of NTS infection in children. While this study provides valuable insights, several limitations should be noted. First, the stratified analysis by follow-up duration suggests that the increased risk of NTS infection is most pronounced during the first 1 to 4 years after appendectomy, with a statistically significant elevated hazard. Although the aHR remains above unity beyond 5 years postsurgery, this elevation is not statistically significant, suggesting the possibility of a diminishing risk over time. This pattern highlights the need for further research into the long-term immune adaptation and potential recovery processes following appendectomy. Second, beyond rural or urban location and young age, additional factors predisposing to NTS infection include underlying medical conditions. For example, transfusion-naive thalassemia and anemia have been linked to increased risk of NTS hospitalization or bacteremia in children,^26,47^ while chronic illnesses and bowel resection may alter gut immunity and elevate risk.^48^ Socioeconomic disadvantage is also associated with higher incidence, especially among young children.^49^ Household exposure and contaminated food or water^50^ are strong risk factors. These variables may represent residual confounding if incompletely adjusted in administrative datasets. To address these limitations, future studies should incorporate more granular clinical and environmental data, possibly through linkage with microbiological, laboratory, or public health surveillance systems.

## Conclusions

In this nationwide cohort study of Taiwanese children, appendectomy was associated with an increased risk of NTS infection. While biologically plausible given the appendix’s immunologic role, this finding warrants cautious interpretation and confirmation in diverse populations. These results underscore the need for continued surveillance and preventive care following appendectomy and for further research to elucidate underlying mechanisms and develop targeted strategies for vulnerable groups.

## Notes

Supplement 1. eTable 1. Multivariable Analysis of Factors Associated Nontyphoidal Salmonella Infection eTable 2. Conditional Cox Proportional Hazards Regression Analysis Stratified by Matched Sets

Supplement 2. Data Sharing Statement
