# Supplementary material for: Appendectomy and Risk of Nonyphoidal Salmonella Infection in Children
Source: JAMA Netw Open. 2026 Jan 23;9(1):e2555278. doi: 10.1001/jamanetworkopen.2025.55278 (PMC12831160; doi:10.1001/jamanetworkopen.2025.55278)
Supplement: Supplement 1. — eTable 1. Multivariable Analysis of Factors Associated Nontyphoidal Salmonella Infection eTable 2. Conditional Cox Proportional Hazards Regression Analysis Stratified by Matched Sets [file jamanetwopen-e2555278-s001.pdf]

Supplemental Online Content

Guo J-Y, Lin W-S, Lin C-H, Wu M-C. Appendectomy and risk of non-typhoidal salmonella infection in children. *JAMA Netw Open*. 2026;9(1):e2555278. doi:10.1001/jamanetworkopen.2025.55278

eTable 1. Multivariable Analysis of Factors Associated Nontyphoidal Salmonella Infection

eTable 2. Conditional Cox Proportional Hazards Regression Analysis Stratified by Matched Sets

This supplemental material has been provided by the authors to give readers additional information about their work.

© 2026 Guo J-Y et al. *JAMA Network Open*.

eTable 1. Multivariable Analysis of Factors Associated Nontyphoidal Salmonella Infection

Characteristic	Cox Proportional Hazards Model				Cox Proportional Hazards Model				Conditional Cox Proportional Hazards Model			
	Crude HR	95%CI		P-value	Adjusted HR	95%CI		P-value	Adjusted HR	95%CI		P-value
**appendectomy operation group**												
No	1.00				1.00				1.00			
Yes	1.65	1.23	2.23	.001	1.58	1.17	2.13	.003	1.58	1.17	2.14	.003
**Age**									-			
<5	15.63	7.26	33.64	<.001	14.88	6.88	32.18	<.001				
5-9	2.65	1.21	5.80	.015	2.56	1.17	5.62	.019				
10-14	1.40	0.61	3.21	.431	1.41	0.61	3.23	.422				
**≥15**	1.00				1.00							
**Gender**									-			
Female	1.00				1.00							
Male	1.35	1.00	1.82	.047	1.40	1.04	1.89	.027				
**Family income**												
$≤18780	1.00				1.00				1.00			
$18781-27600	1.06	0.77	1.46	.730	0.99	0.72	1.37	.963	0.96	0.69	1.33	.808
$27601-42000	0.88	0.59	1.31	.519	0.83	0.55	1.25	.374	0.86	0.57	1.30	.465
$>42000	0.93	0.56	1.53	.768	0.84	0.51	1.40	.504	0.91	0.54	1.54	.734
**Urbanization**												
Urban	1.00				1.00				1.00			
Suburban	1.13	0.76	1.67	.555	1.07	0.72	1.60	.726	1.10	0.73	1.64	.655
Rural	1.12	0.83	1.52	.457	1.10	0.81	1.50	.540	1.08	0.79	1.47	.644
**Comorbidity**												
**Asthma**												
No	1.00				1.00				1.00			

© 2026 Guo J-Y et al. *JAMA Network Open*.

Yes	0.73	0.53	1.02	.066	0.95	0.67	1.35	.771	0.93	0.65	1.33	.695
**Atopic dermatitis**												
No	1.00				1.00				1.00			
Yes	1.42	1.05	1.91	.023	1.46	1.08	1.98	.015	1.57	1.15	2.16	.005
**Congenital heart anomaly**												
No	1.00				1.00				1.00			
Yes	0.69	0.26	1.85	.460	0.67	0.25	1.81	.429	0.64	0.24	1.76	.389
**Congenital gastrointestinal anomaly disease**												
No	1.00				1.00				1.00			
Yes	0.97	0.24	3.89	.963	0.79	0.19	3.19	.737	0.73	0.18	3.02	.660
**Chronic liver diseases**												
No	1.00				1.00				1.00			
Yes	0.96	0.24	3.85	.951	1.20	0.30	4.87	.795	1.23	0.30	5.10	.780
**Constipation**												
No	1.00				1.00				1.00			
Yes	0.90	0.63	1.29	.562	1.01	0.70	1.45	.976	1.06	0.73	1.54	.773
**Antibiotic (before 1 year)**												
No	1.00				1.00				1.00			
Yes	2.02	1.52	2.70	<.001	1.47	1.09	1.97	.011	1.46	1.08	1.98	.014

© 2026 Guo J-Y et al. *JAMA Network Open*.

eTable 2. Conditional Cox Proportional Hazards Regression Analysis Stratified by Matched Sets

Characteristic	Model 1			Model 2			Model 3			Model 4		
	Adjusted HR	95%CI	P-value	Adjusted HR	95%CI	P-value	Adjusted HR	95%CI	P-value	Adjusted HR	95%CI	P-value
**appendectomy operation group**												
No	1.00			1.00			1.00			1.00		
Yes	1.66	1.23 2.24	.001	1.66	1.23 2.24	.001	1.64	1.22 2.21	.001	1.64	1.21 2.21	.001
**Age**												
<5	1.00			1.00			1.00			1.00		
5-9	15.64	7.27 33.67	<.001	15.66	7.28 33.71	<.001	15.87	7.37 34.17	<.001	15.92	7.37 34.38	<.0001
10-14	2.65	1.21 5.80	.015	2.62	1.20 5.75	.016	2.66	1.21 5.83	.015	2.62	1.20 5.75	.016
**≥15**	1.40	0.61 3.21	.431	1.38	0.60 3.18	.445	1.40	0.61 3.21	.432	1.38	0.60 3.17	.449
**Gender**												
Female				1.00			1.00			1.00		
Male				1.41	1.05 1.90	.022	1.42	1.05 1.91	.022	1.41	1.05 1.90	.024
**Family income**												
$≤18780							1.00			1.00		
$18781-27600							1.01	0.73 1.39	.970	1.00	0.72 1.37	.975
$27601-42000							0.84	0.56 1.26	.398	0.83	0.55 1.24	.357
$>42000							0.85	0.51 1.41	.521	0.83	0.50 1.38	.469
**Urbanization**												
Urban							1.00			1.00		
Suburban							1.07	0.72 1.60	.724	1.08	0.73 1.60	.713
Rural							1.10	0.81 1.49	.551	1.10	0.81 1.50	.526
**Comorbidity**												

© 2026 Guo J-Y et al. *JAMA Network Open*.

**Asthma**				
No	1.00			
Yes	0.99	0.70	1.40	.950
**Atopic dermatitis**				
No	1.00			
Yes	1.50	1.10	2.03	.010
**Congenital heart anomaly**				
No	1.00			
Yes	0.69	0.26	1.85	.457
**Congenital gastrointestinal anomaly disease**				
No	1.00			
Yes	0.81	0.20	3.26	.763
**Chronic liver diseases**				
No	1.00			
Yes	1.25	0.31	5.07	.752
**Constipation**				
No	1.00			
Yes	1.04	0.72	1.50	.844
**Antibiotic (before 1 year)**				
No				
Yes				

© 2026 Guo J-Y et al. *JAMA Network Open*.
