# Supplementary material for: Appendectomy and Risk of Nonyphoidal Salmonella Infection in Children
Source: JAMA Netw Open. 2026 Jan 23;9(1):e2555278. doi: 10.1001/jamanetworkopen.2025.55278 (PMC12831160; doi:10.1001/jamanetworkopen.2025.55278)
Supplement: Supplement 2. — Data Sharing Statement [file jamanetwopen-e2555278-s002.pdf]

Data Sharing Statement

Guo. Appendectomy and Risk of Nonyphoidal Salmonella Infection in Children. *JAMA Netw Open*. Published January 23, 2026. doi:10.1001/jamanetworkopen.2025.55278

Data
Data available: Yes
Data types: Deidentified participant data
How to access data: none
When available: With publication

Supporting Documents
Document types: None

Additional Information
Who can access the data: None
Types of analyses: None
Mechanisms of data availability: None
